# Endobronchial Lipoma: A Rare Benign Tumor Managed by Minimally Invasive Bronchoscopic Excision

**DOI:** 10.7759/cureus.90623

**Published:** 2025-08-20

**Authors:** Ashwin Kailash, Yuvarajan Sivaganame

**Affiliations:** 1 Department of Respiratory Medicine, Sree Balaji Medical College and Hospital, Chennai, IND; 2 Department of Respiratory Medicine, Sri Manakula Vinayagar Medical College and Hospital, Puducherry, IND

**Keywords:** bronchoscopic resection, cryoprobe, endobronchial lipoma, lobar collapse, snare excision

## Abstract

Endobronchial lipoma is a rare, benign tumor of the tracheobronchial tree, often misdiagnosed due to overlapping features with other pulmonary conditions. The clinical presentation of benign and malignant endobronchial tumors can be indistinguishable, complicating diagnosis. Definitive diagnosis requires histopathological confirmation via bronchoscopy-guided biopsy or excision. Treatment is based on the degree of airway obstruction and the extent of distal lung damage.

We presented a case of a 52-year-old female with bronchial asthma, who was found to have an endobronchial lipoma causing middle and lower lobe collapse. The lesion was successfully treated with bronchoscopic resection using a snare and cryoprobe, avoiding the need for surgical lobectomy.

## Introduction

Benign endobronchial tumors are rare, and it has been reported that approximately 11.2% of endobronchial tumors are benign, with an average annual incidence of 23 new cases and an overall incidence of 2.2% [1]. Among these, endobronchial lipomas are particularly rare - comprising only 0.1%-0.5% of all pulmonary tumors [2]. These lesions typically arise from adipose tissue in the peribronchial or submucosal layers and may remain undiagnosed until they cause significant bronchial obstruction. Their intraluminal growth can lead to atelectasis, obstructive pneumonitis, and even irreversible changes like bronchiectasis [2]. Differentiating them from malignant tumors is essential but challenging. In this article, we report a case of endobronchial lipoma arising from the right bronchus intermedius, presenting with lobar collapse and successfully managed via endoscopic resection, thereby preventing major surgery such as lobectomy.

## Case presentation

A 52-year-old female homemaker with a known history of bronchial asthma and type 2 diabetes mellitus presented with a three-week history of progressively worsening dyspnea and productive cough with mucopurulent expectoration. Despite multiple courses of inhaled bronchodilators and antibiotics prescribed in the outpatient setting, her symptoms persisted. During admission, she experienced increasing breathlessness on exertion and reduced exercise tolerance, prompting further evaluation.

On examination, her vital signs were stable: SpO₂ was 96% on 4 L/min oxygen, blood pressure was 120/80 mmHg, respiratory rate was 26 breaths/min, and heart rate was 105 bpm. Auscultation revealed decreased breath sounds over the right interscapular, infrascapular, and infra-axillary areas, with scattered wheezes in other lung fields.

**Figure 1:**
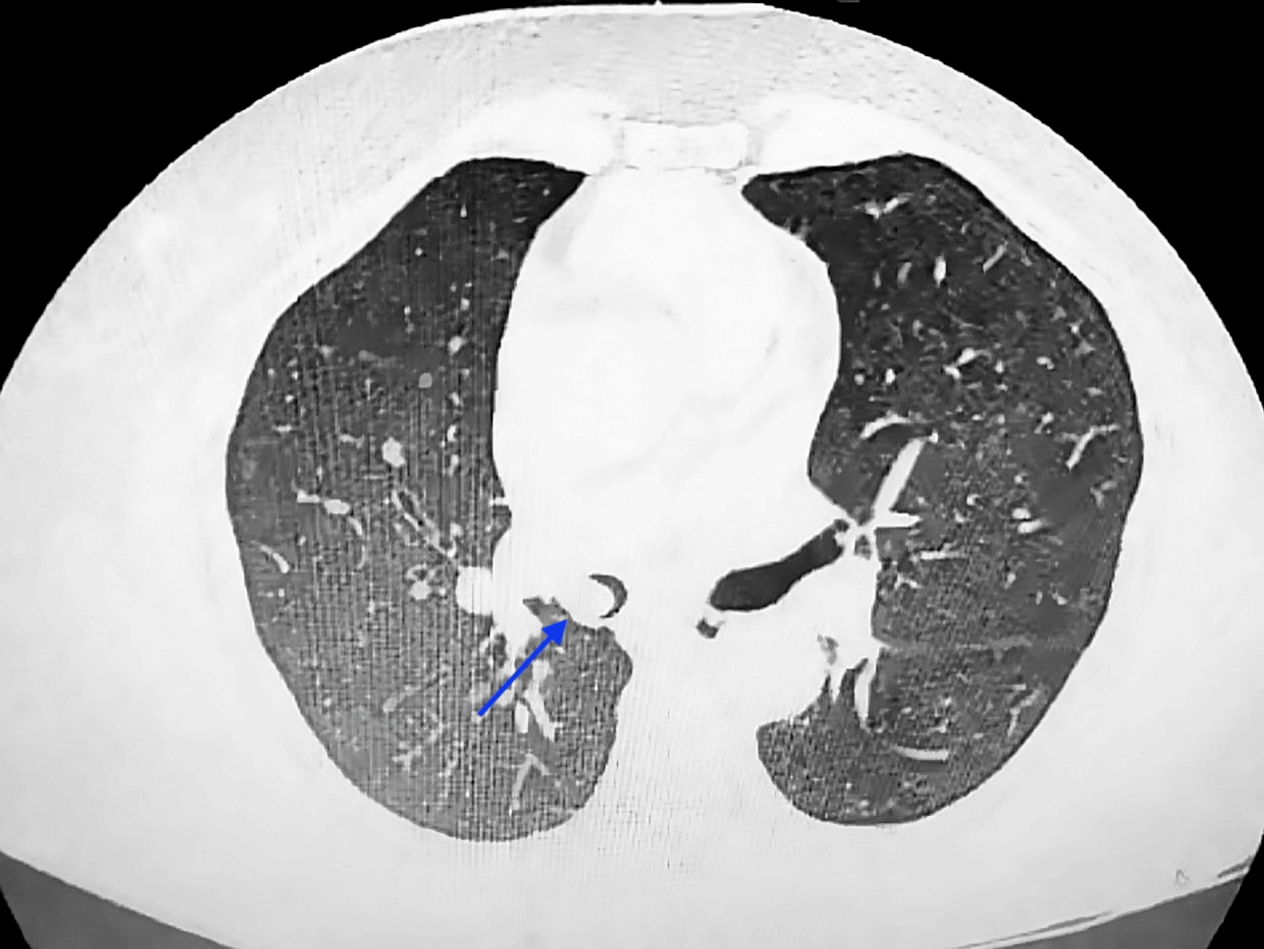
Axial HRCT of the thorax showing an endobronchial lesion in the right main bronchus Lung window HRCT axial view shows a homogeneous fat-density lesion (HU-65) in the right main bronchus (blue arrow), causing partial lumen obstruction. HRCT, High-Resolution Computed Tomography

**Figure 2:**
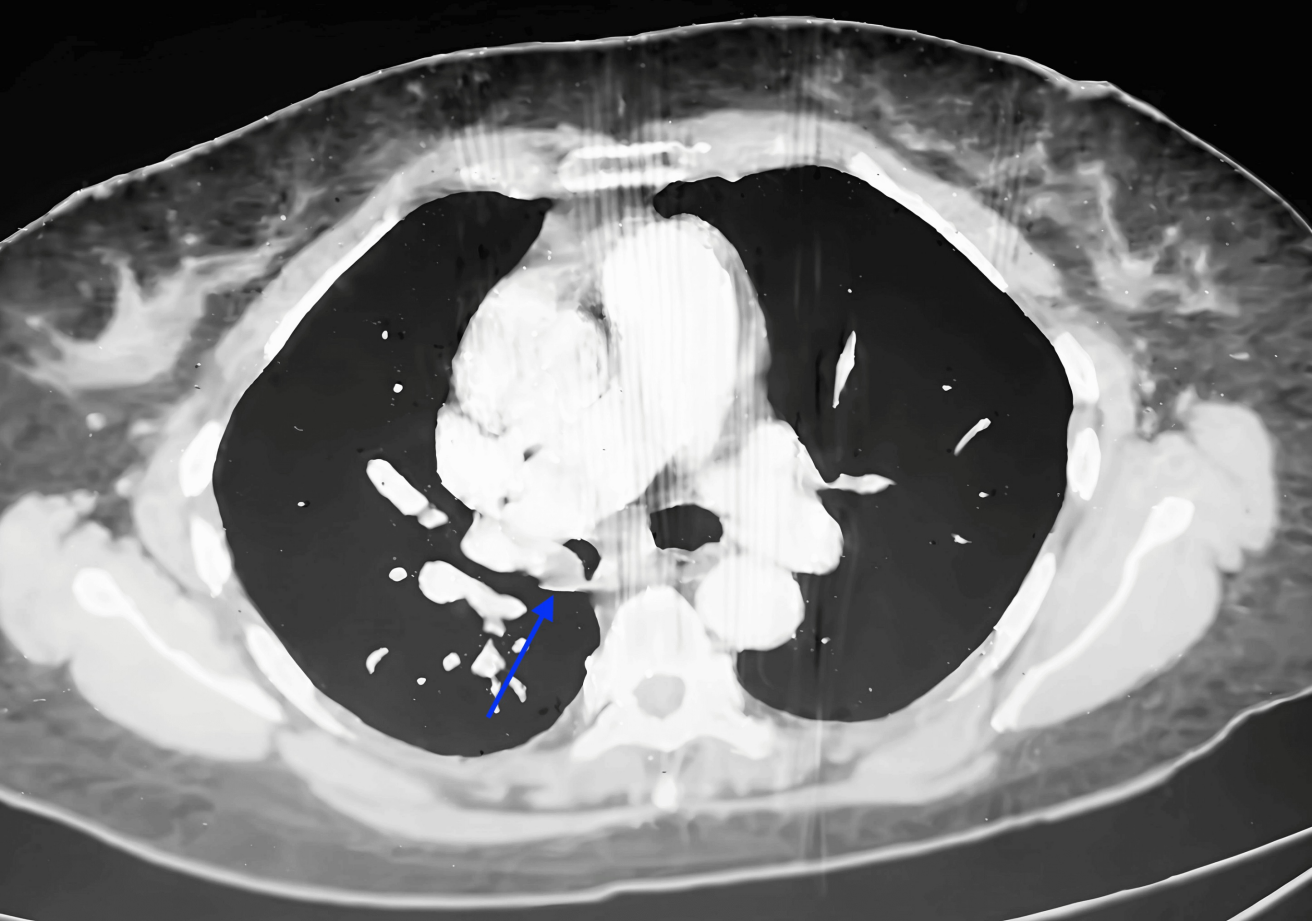
Axial CT of the thorax (mediastinal window) reveals an endobronchial obstruction in the right main bronchus Axial CT of the thorax (mediastinal window) demonstrates a well-defined lesion (14 × 18 mm) (blue arrow) within the right main bronchus, causing near-complete endobronchial obstruction. CT, Computed Tomography

**Figure 3:**
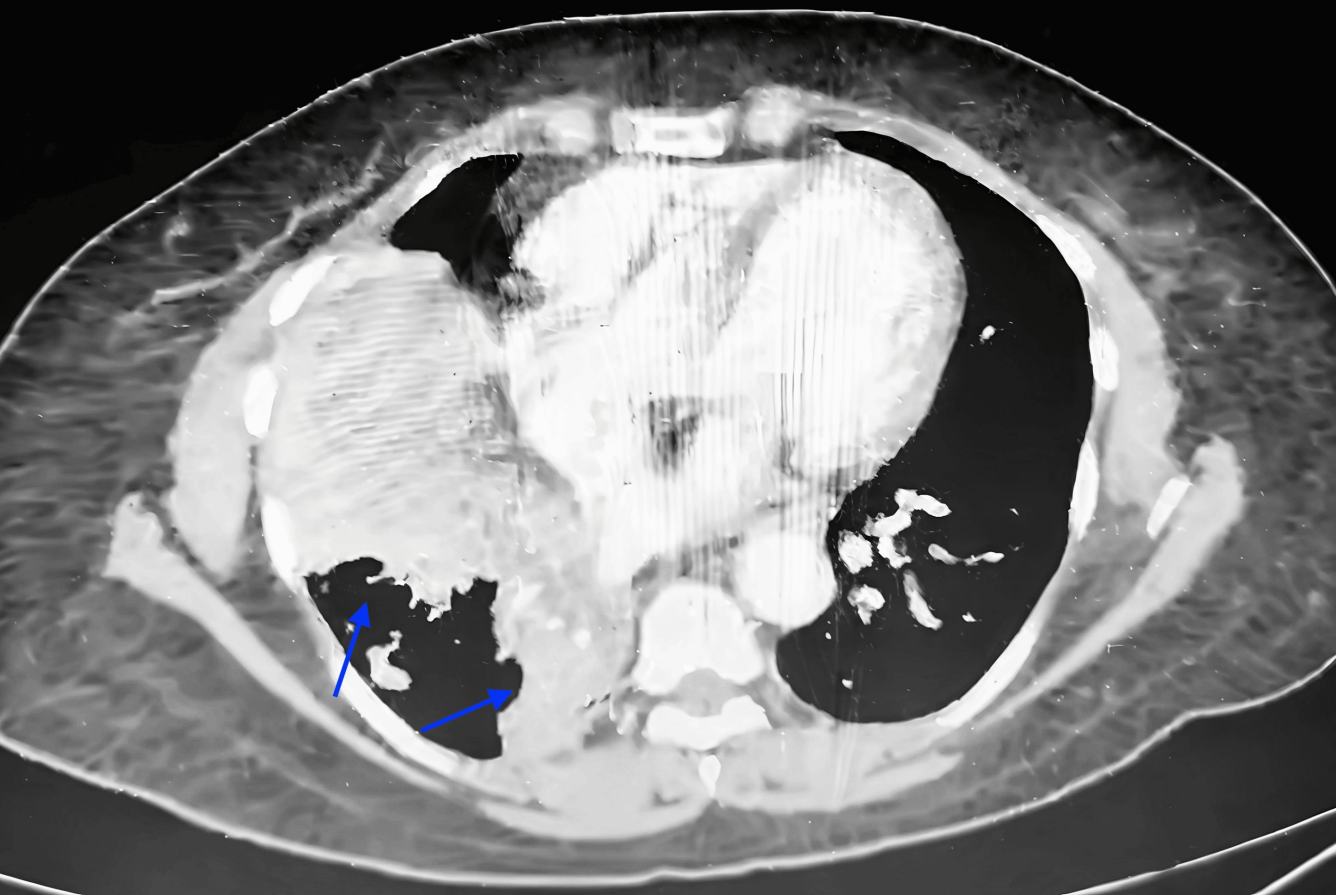
Axial CT of the thorax showing partial collapse of the right lower lobe secondary to endobronchial obstruction (blue arrows) CT, Computed Tomography

Chest X-ray showed homogeneous opacity in the right lower zone, with blunting of the costophrenic angle. Computed tomography (CT) of the chest revealed segmental collapse of the right middle and lower lobes, likely secondary to endobronchial obstruction (presumed mucus plug) (Figures 1-3).

**Figure 4:**
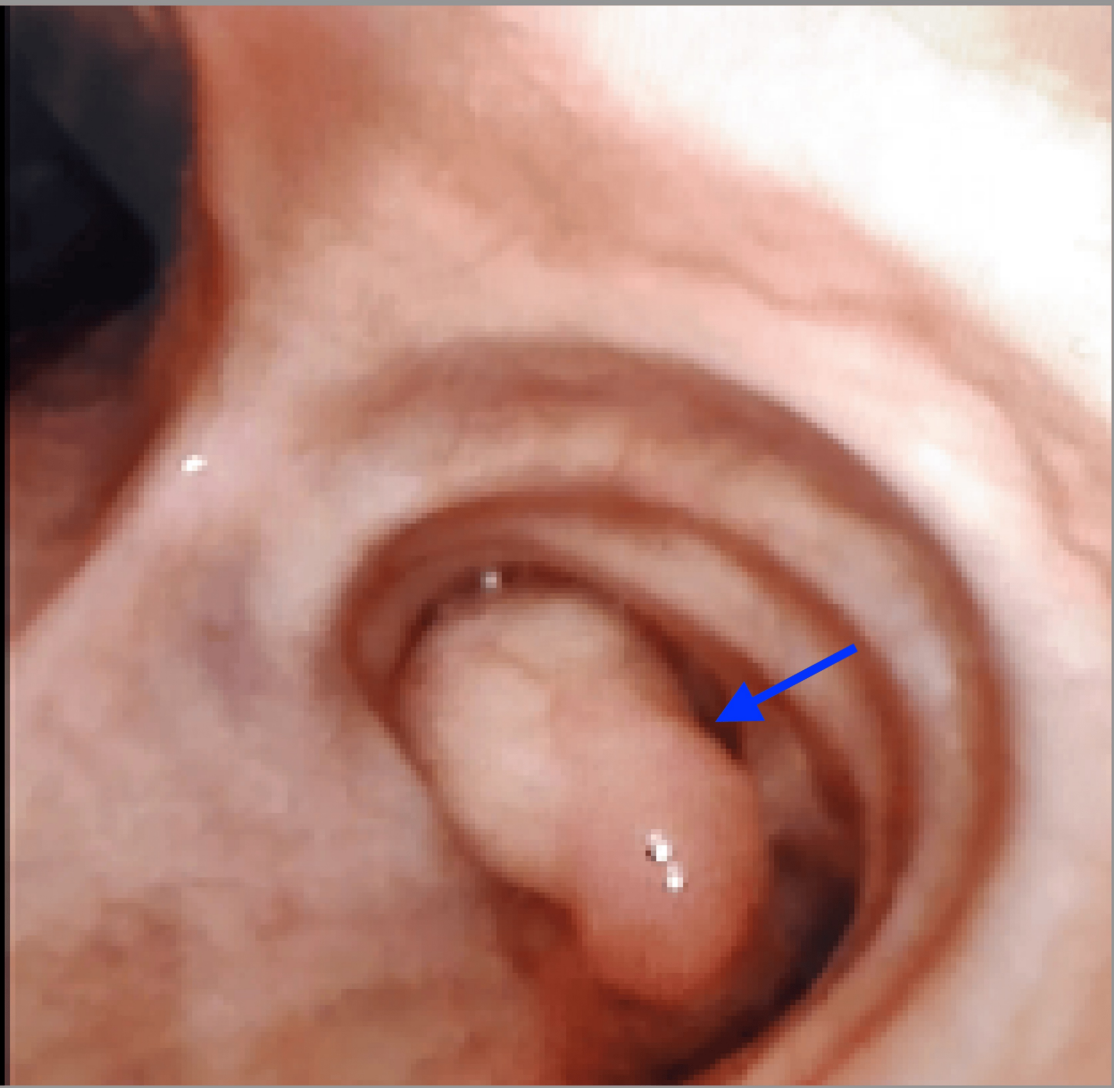
Bronchoscopic view showing a smooth endobronchial mass obstructing the right bronchus intermedius Flexible bronchoscopy reveals a smooth, well-circumscribed, pedunculated endobronchial lesion (blue arrow), causing significant obstruction.

Flexible bronchoscopy under local anesthesia demonstrated a smooth, polypoid mass completely obstructing the right bronchus intermedius (Figure 4). The scope could not be negotiated beyond the lesion; therefore, resection under general anesthesia was planned.

**Figure 5:**
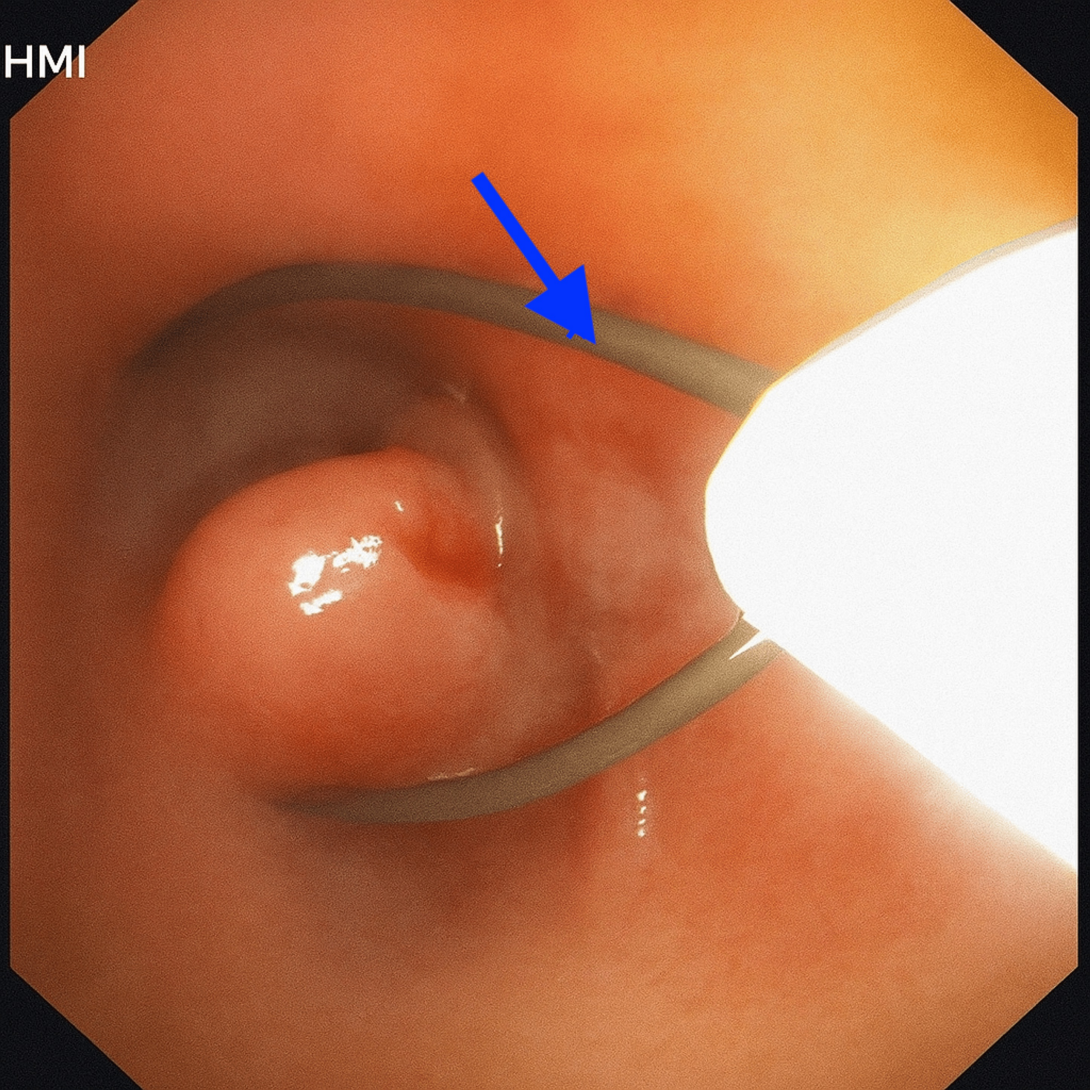
Bronchoscopic image showing a 15 mm snare deployed around an endobronchial mass Flexible bronchoscopic view demonstrating the use of a 15 mm snare looped around the pedunculated endobronchial mass arising from the right lower lobe bronchus (blue arrow). The snare was used for mechanical resection under vision as part of the minimally invasive therapeutic approach.

**Figure 6:**
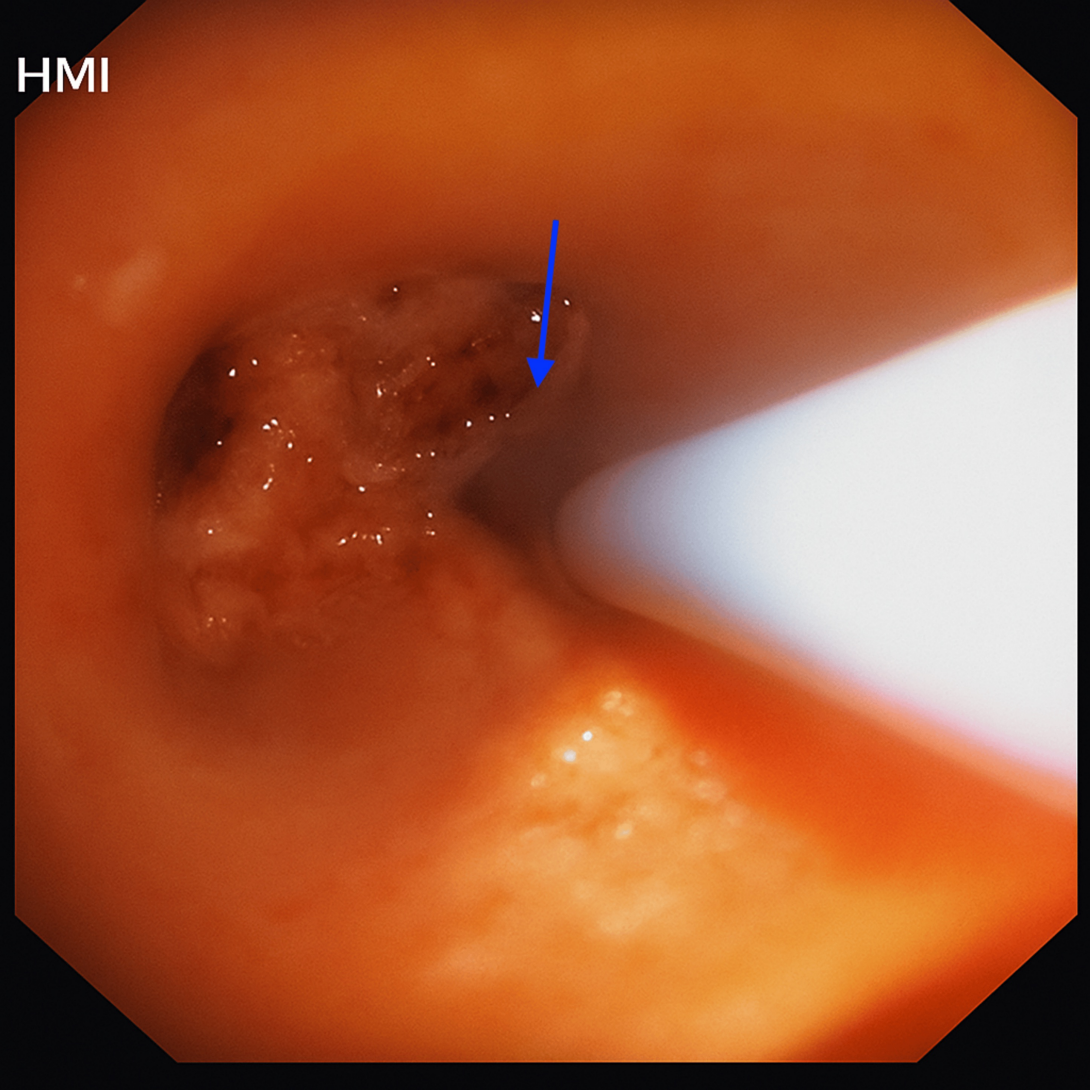
Bronchoscopic image shows a 1.1 mm cryoprobe application to extract residual tumour tissue Flexible bronchoscopy view demonstrating the application of a cryoprobe (blue arrow) to the residual tissue at the base of the previously resected endobronchial mass. Cryoextraction was performed to ensure complete removal and to obtain additional tissue for histological analysis.

**Figure 7:**
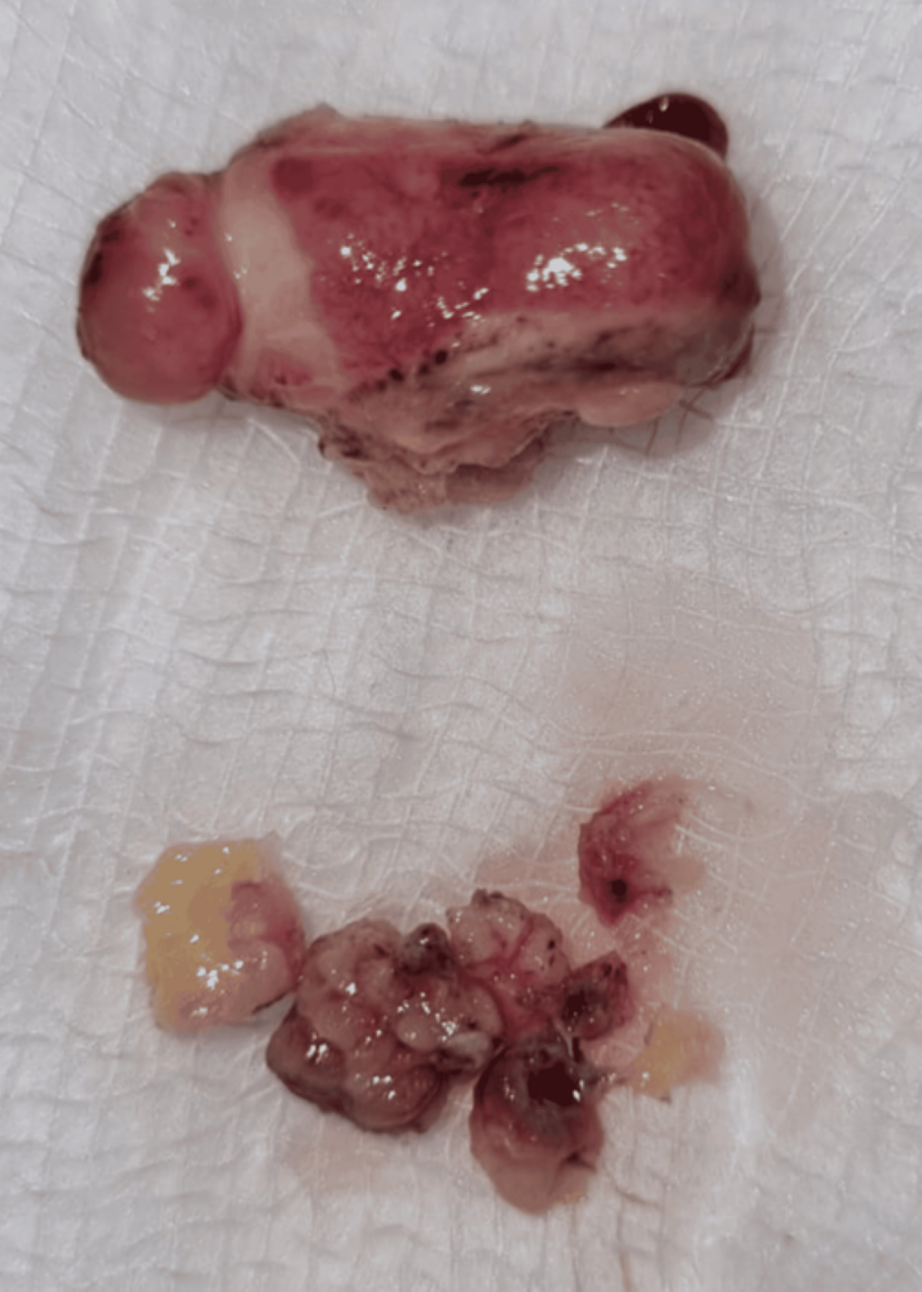
Gross specimen of the excised lipoma with adherent tissue Macroscopic view of the excised lesion shows a well-circumscribed, ovoid mass consistent with a lipomatous tumor.

**Figure 8:**
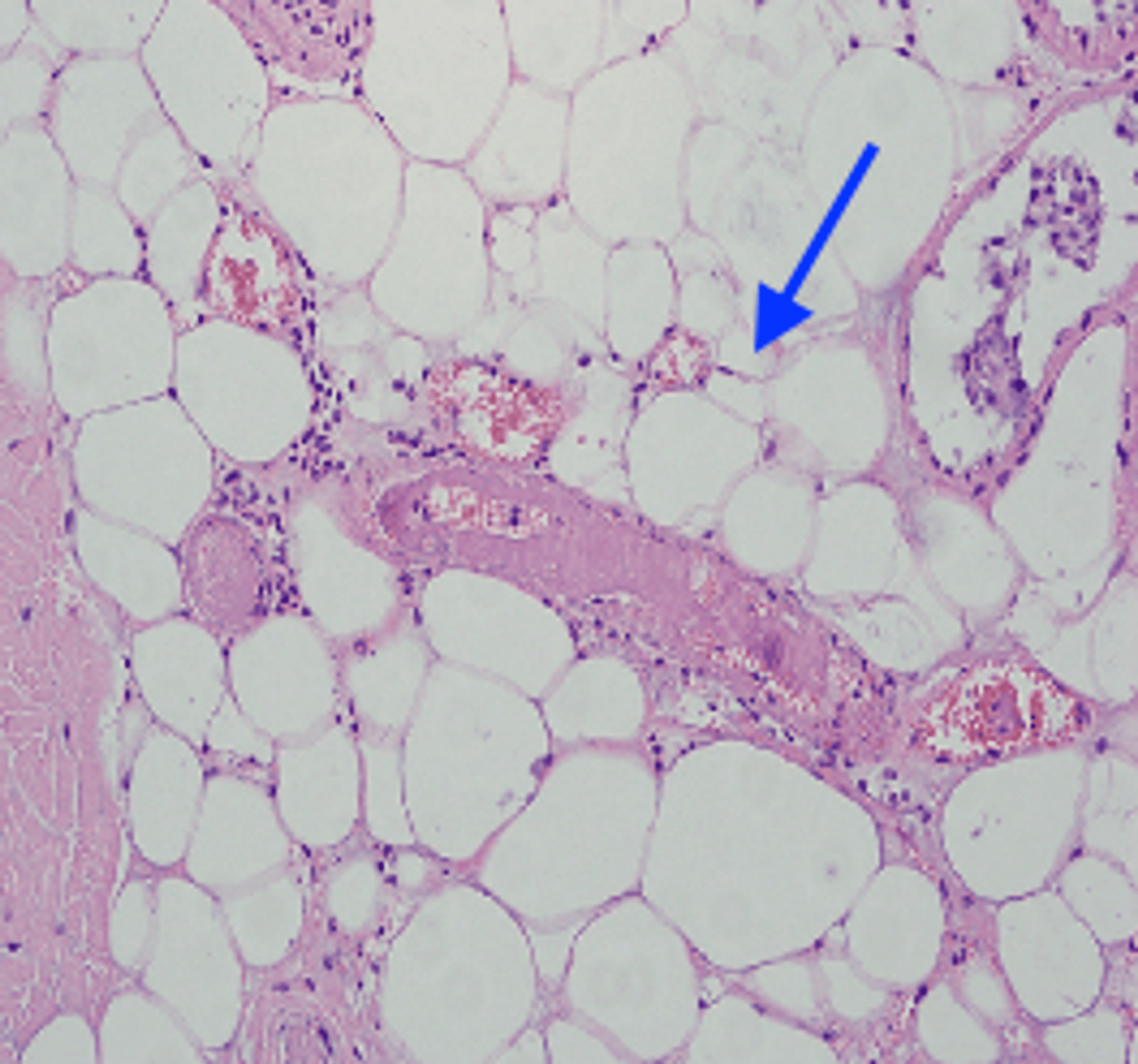
Histopathology image shows mature adipocytes with fibrous septa Hematoxylin and eosin (H&E) stained section under 40x magnification reveals sheets of mature, unvacuolated adipocytes separated by thin fibrous septa (blue arrow), without cellular atypia, lipoblasts, or necrosis, confirming the diagnosis of benign lipoma.

The procedure was performed under general anaesthesia, with the patient in a supine position. A rigid bronchoscope was introduced, ensuring airway protection and adequate oxygenation throughout. On visualization, a smooth, pedunculated mass was noted obstructing the right bronchus intermedius. The lesion was grasped and excised en bloc using a 15 mm electrosurgical snare (Figure 5). Following excision, residual tissue fragments were carefully removed using a 1.1 mm cryoprobe (Figure 6) to ensure complete clearance of the airway lumen. Hemostasis was achieved with minimal cautery, and no intraoperative bleeding or airway compromise occurred. The excised specimen was then sent for histopathological examination (Figure 7). The examination revealed lobules of mature adipocytes with uniform nuclei and abundant clear cytoplasm, separated by thin fibrous septa (Figure 8). No cellular atypia, necrosis, or mitotic activity was noted, confirming the diagnosis of endobronchial lipoma.

**Figure 9:**
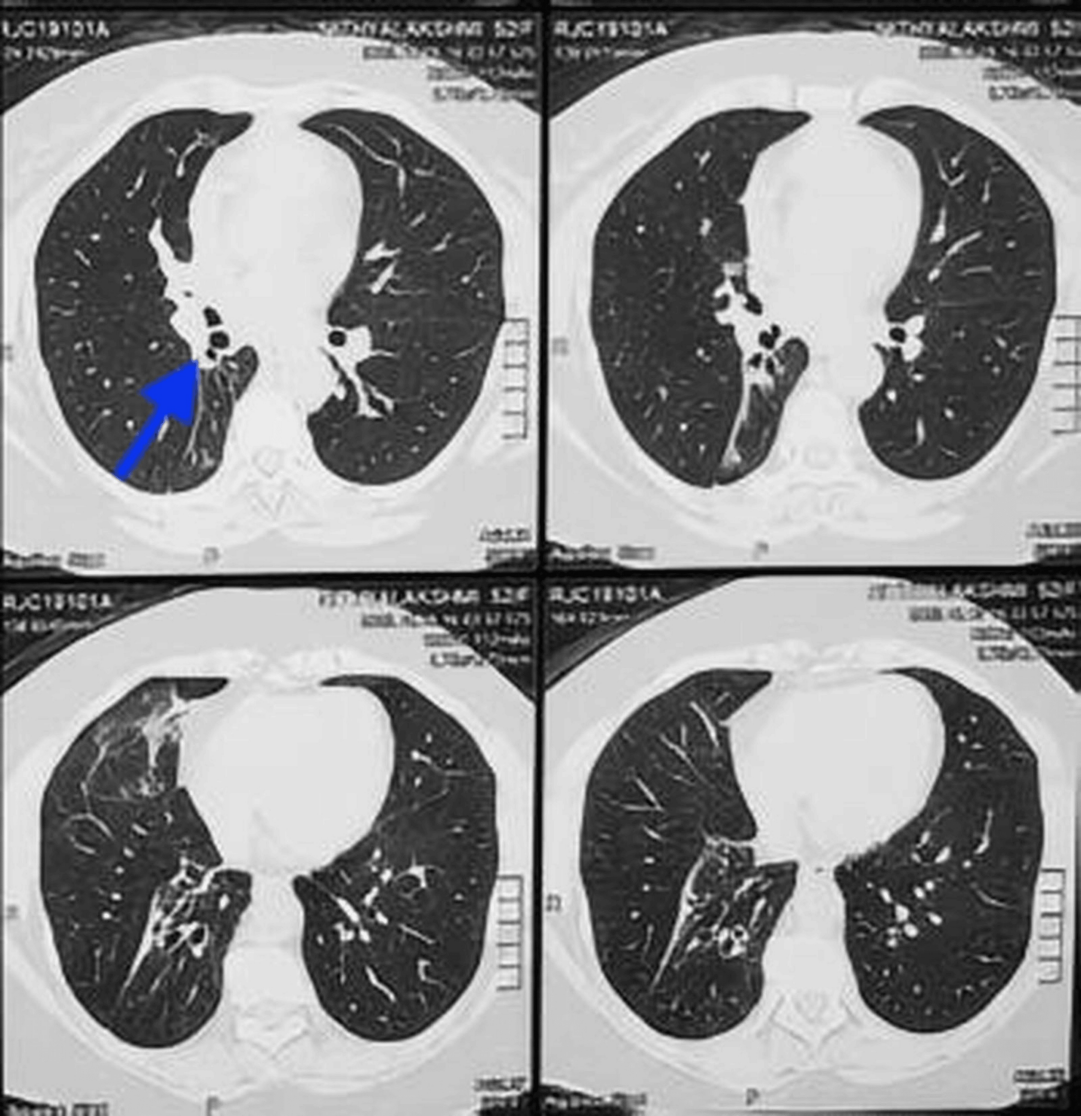
Postoperative CT images showing the re-expansion of the previously collapsed lobe (blue arrow) Axial HRCT thorax images taken post-endobronchial resection demonstrate complete re-expansion of the right lower lobe. No residual lesion or post-obstructive consolidation is seen. Lung parenchyma appears aerated and well preserved. HRCT, High-Resolution Computed Tomography

**Figure 10:**
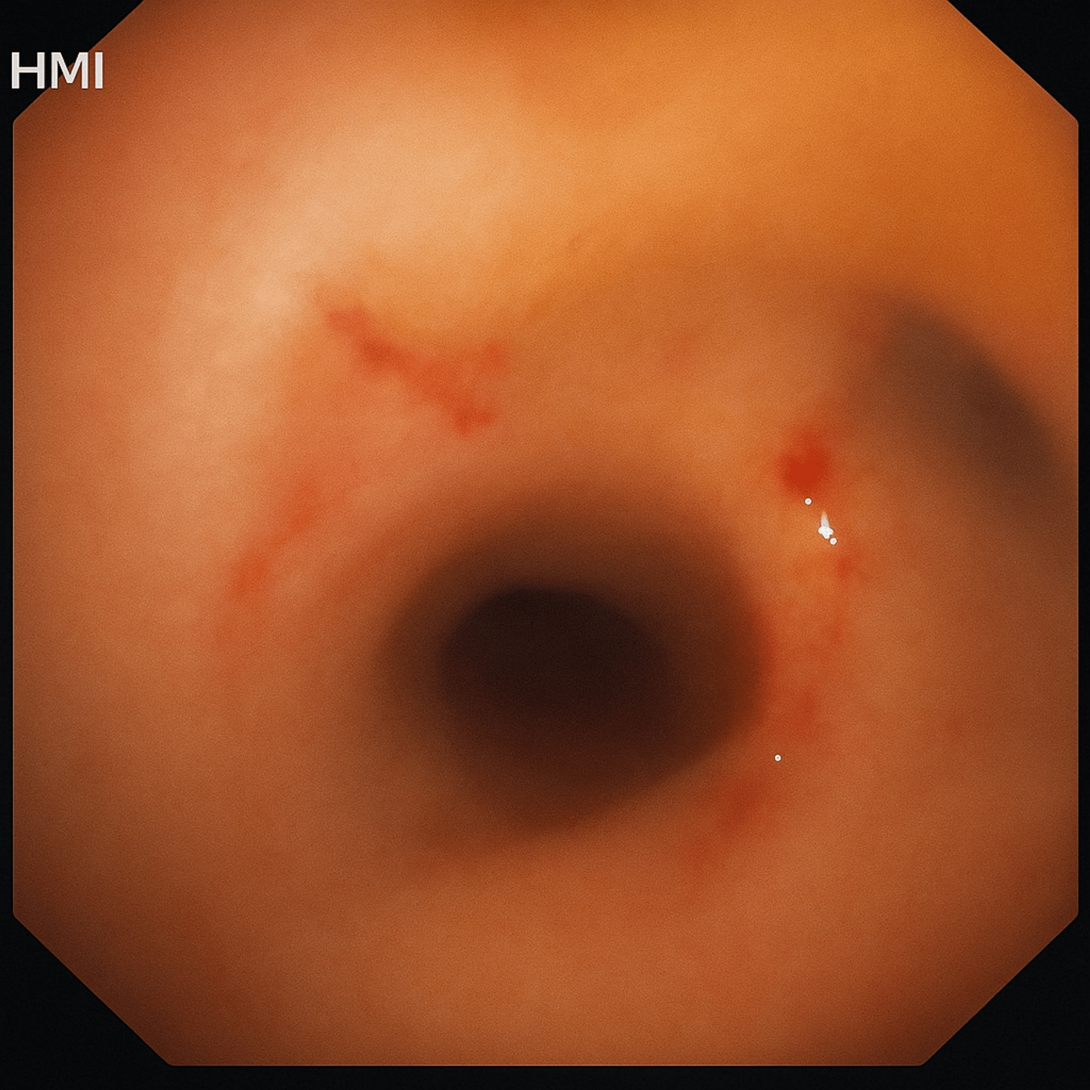
Post-procedure bronchoscopic image shows a patent bronchus intermedius with minimal healing fibrotic changes Follow-up flexible bronchoscopy (after six weeks) demonstrates a widely patent bronchus intermedius with no residual endobronchial lesion. Minimal mucosal irregularity and focal fibrotic changes were noted.

The postoperative course was uneventful. The patient experienced significant symptomatic improvement. Continuous monitoring was performed intraoperatively to detect potential complications such as bleeding, hypoxemia, or airway compromise. Minimal bleeding occurred during excision and was controlled with electrocautery. No airway injury, hypoxia, or cardiovascular instability was observed. The patient was extubated safely and monitored in the recovery unit with supplemental oxygen for 24 hours. Follow-up imaging showed complete resolution of lobar collapse (Figure 9). Flexible bronchoscopy performed six weeks later revealed a widely patent bronchus intermedius with no residual endobronchial lesion. Minimal mucosal irregularity and focal fibrotic changes were noted (Figure 10), confirming satisfactory healing with no early recurrence or complications.

## Discussion

Most endobronchial tumors are malignant; benign variants like lipomas are rare and often underdiagnosed [3,4]. Intraluminal lipomas can cause obstruction of the bronchial tree, resulting in cough, expectoration, wheezing, and dyspnea [5]. Lipomas can arise from intrapulmonary adipose tissue. Although they predominantly consist of mature fat cells, other components, such as cartilage or bone, may occasionally be found [6]. They are most commonly seen in middle-aged males, with a reported male-to-female ratio of 45:7 [7]. These tumors cause mechanical airway obstruction, leading to distal atelectasis, infection, and occasionally bronchiectasis [8].

Symptoms are often non-specific and may mimic bronchial asthma or chronic obstructive pulmonary disease (COPD) [9]. In our patient, a misdiagnosis of asthma delayed the correct diagnosis. Obesity and smoking are reported as potential - but not definitively established - risk factors [10].

Chest imaging may show indirect signs, such as atelectasis or consolidation. CT scans may reveal a fat-density lesion within the bronchial lumen, aiding in diagnosis. A homogeneous fat attenuation between -30 and -70 Hounsfield Units (HU) is characteristic of lipoma [11]. However, definitive diagnosis depends on histological confirmation. Biopsy via bronchoscopy has a diagnostic yield of only 50%, partly due to the slippery surface and submucosal nature of the tumor [12].

These tumors are lined with normal respiratory mucosa and may show squamous metaplasia. Incomplete or non-representative biopsies can lead to diagnostic uncertainty, necessitating complete excision [13]. Early bronchoscopic intervention can prevent the need for more extensive surgical procedures, like lobectomy or pneumonectomy [14].

Our case illustrates that minimally invasive bronchoscopic resection with snare and cryoprobe is a safe and effective option for both diagnosis and treatment. The patient avoided surgery, and her clinical and radiologic response was excellent.

## Conclusions

Endobronchial lipomas, although rare, should be considered in patients with unexplained, persistent pulmonary symptoms or lobar collapse, especially when unresponsive to conventional treatments like asthma management. CT features may suggest the diagnosis, but definitive identification requires histopathologic confirmation. Bronchoscopic resection is both diagnostic and therapeutic and should be considered the first-line approach when technically feasible.
